# Undergraduate nursing students’ experiences of becoming a professional nurse: a longitudinal study

**DOI:** 10.1186/s12912-022-01002-0

**Published:** 2022-08-06

**Authors:** Susanne Lundell Rudberg, Margareta Westerbotn, Taina Sormunen, Max Scheja, Hanna Lachmann

**Affiliations:** 1 Department of Learning, Informatics, Management and Ethics, Karolinska Institutet, 171 77 Stockholm, Sweden; 2 Department of Health Promoting Science, Sophiahemmet University, P. O. Box 5605, 114 86 Stockholm, Sweden; 3 Department of Nursing Science, Sophiahemmet University, P. O. Box 5605, 114 86 Stockholm, Sweden; 4 Department of Clinical Science and Education, Södersjukhuset, Karolinska Institutet, 118 83 Stockholm, Sweden; 5 Department of Education of Stockholm University, 106 91 Stockholm, Sweden

**Keywords:** Students, Nursing, Professionalism, Education, Nursing, Professional Competence, Emotions, Qualitative study

## Abstract

**Background:**

During education it is essential for nursing students to develop professionalism in nursing. Nurses are placed in situations based on trust, and it is crucial that their patients have confidence in them to provide professional and safe care. A key period in nursing students’ development of a professionalism occurs during training when students gain knowledge and skills that separate nurses as professional healthcare workers from laypeople. The purpose of this study was to investigate nursing students’ experiences of professional competence development during education.

**Methods:**

A longitudinal study was carried out using qualitative content analysis with a manifest inductive approach. Thirty-four students enrolled in a Swedish three-year nursing program, from August 2015 to January 2017 were interviewed on four occasions.

**Results:**

The results revealed that students’ professional role developed gradually. The students’ started their education with dreams and a naive understanding of the profession, but their understanding of the complexity of the nursing profession gradually evolved. Students became theoretically equipped at the university and developed clinical skills through practice. Students’ focus went from mastering medical technology to a more holistic approach. Before graduating, students felt ready but not fully trained.

**Conclusions:**

Our findings indicate a discrepancy between the content of the theoretical education and the clinical settings since students identified a lack of evidence-based practice. A solid theoretical education before entering clinical training offered students possibilities for reflecting on evidence-based practice and the clinical settings. The realization that there is always potential for professional improvement can be interpreted as an emerging awareness, and development of professionalism. It is clear that students could benefit from increased collaborative work between clinical supervisors and faculty staff at the university.

**Supplementary Information:**

The online version contains supplementary material available at 10.1186/s12912-022-01002-0.

## Background

It is essential to maintain professionalism in the nursing profession [1]. Nurses are placed in situations based on trust, and it is crucial that the persons depending on these professionals have confidence in them to provide professional and safe care [1]. A key period in nursing students’ development of a professionalism occurs during training when they gain knowledge and skills that separate nurses as professional healthcare workers from laypeople [2]. However, there is limited theoretical knowledge of the aspects that constitute and drive the development of nursing students’ professional competence.

### Professionalism and competence

The term ‘professionalism’ is used globally to describe professions with a nonspecific focus. There is no simple, generalizable definition of the multidimensional concept of professionalism, or a simple way of assessing it [3]. When applied to nursing, professionalism is associated with behaviors such as a belief in public service, autonomy and self-regulation, and a sense of vocation [1]. So the concept of professionalism is complex and maintaining professionalism is essential in the nursing profession [3]. Apart from being time specific and related to specific contexts, nursing students’ competencies, domains and levels vary by professional assignment, description, and country [4]. The governance of nursing education varies globally, and is controlled by national regulations [5]. It has become common to use a holistic view in defining nurse competencies, including behavior statements reflecting the skills, knowledge, attitudes, and judgments required for effective performance in the nursing profession [6, 7]. Professionalism in healthcare is associated with a wide range of benefits, for example staff displaying higher levels of professional attitudes also seem to behave more professionally [8], and increased safety for patients [9]. To integrate quality into nursing education a framework has been developed comprising six nurse core competencies; person-centered care, evidence-based practice, teamwork and collaboration, safety, quality improvement and informatics [10].

### Nursing students’ journey towards professionalism

Dr Benner describes the concept of professional development in the nursing profession as evolving from novice to expert in five stages: novice, advanced beginner, competent proficient and expert [11]. In health care, the learning process, and also the socialization to develop a professional identity, have often meant adopting standards and norms of the professional group [12, 13]. Nursing students are socialized towards professionalism during training, being required to integrate the attributes of professionalism in their routine practice [1]. Students are motivated to learn when they feel included in the clinical environment, while experiences of exclusion and lack of belonging influences negatively on motivation to learn [14]. Deliktas et al. [15] found that undergraduate nursing students’ approach to the nursing profession is associated with humanism, also identified as conscience, coupled with an ambition to touch people’s lives. It requires great effort from the students together with substantial support from teachers and supervisors to develop a comfortable professional identity upon graduation [16]. Students’ journey to becoming an RN has been investigated previously [17–20]. However, longitudinal studies of students’ experiences of professional development throughout the whole education are scarce.

## Methods

### Aim

The present study aimed at investigating nursing students’ experiences of professional competence development during education.

#### Design

This study applied a descriptive longitudinal design involving qualitative content analysis with a manifest inductive approach.

#### Settings and participants

Thirty-four students enrolled in a Swedish three-year nursing program, starting from August 2015 to January 2017, were followed longitudinally. The group comprised 28 women and six men, a distribution coherent with national statistics of students in health care in HE [21]. In respect of age, students ranged from 20 to 51 on enrolment. Fourteen students had previously attended HE, and ten had completed a university degree in another subject. Twelve reported living in a single household and eight had children of their own, whilst ten students shared a household with up to three children.

#### Data collection

**Fig. 1:**
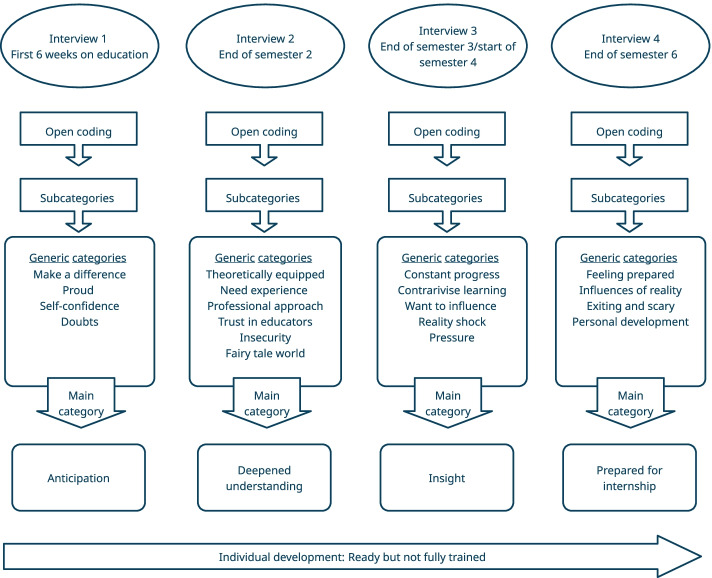
Overview of methods including generic categories, main categories, and overarching themes

A purpose sampling technique was used. During the first week of education, all students enrolled were invited to participate. Information about the purpose of the study was given orally after an introductory lecture and in writing on the university’s learning platform. Students were also informed about whom to contact if they had questions prior to the interviews. Students who signed a written informed consent were invited on four occasions (Fig. 1) to individual, semi-structured interviews carried out in an undisturbed conference room at the university. A total of thirty-four students participated in all interviews. A semistructured interview guide was designed to capture students’ ongoing professional development, including questions about their views of nurse core competencies at interviews two, three and four, (supplementary file). All four interviews were carried out by the first author and lasted five to 40 min. The interviews were audio recorded and transcribed verbatim [22, 23].

The study was approved by the Regional Ethical Review Board, Stockholm (Registration no: 2015/894–31/5).

#### Data analysis

The interview transcripts were read several times making sense of the data as whole [24] and then analyzed using qualitative content analysis with a manifest inductive approach [25, 26]. Data were divided into four chronological data sets, interviews 1–4. Each dataset was read again when conducting open coding, using NVivo software [27]. Identified codes were grouped into sub-categories labelled with a phrase that described the meaning content. Sub-categories were grouped by contextual meaning resulting in 19 generic categories. From this categorization, an abstraction was derived describing one main category of students’ experiences from each interview [28], Fig. 1.

Individual patterns were analyzed to identify and validate shared patterns [29], Table 1.

**Table 1:** Example of individual pattern of professional development

Interview	First interview	Second interview	Third interview	Fourth interview
Student no 8	Left a long career in another area for an interest in people. Indicates a sense of satisfaction in being able to help practically and have a meaningful profession	Learned a lot. Changed view of the nursing profession, the work is more complicated and the responsibility greater than expected. Indicates that it is positive and feels even more convinced that the choice of profession is appropriate	Points out that the clinical reality is not as expected that one has learned about a utopia. Despite this, states that there is a vision of and a goal to be able to provide good healthcare	Feels equipped to take the role of a nurse, still nervous but look forward to soon trying out skills learnt
Student no 26	Several events led to the choice of education. Believes in own ability and expects the education and training to be demanding. Has developed strategies of making an extra effort in theoretical education believing that the clinical training will benefit from that	Has failed exams but describes strong motivation to continue studying. Despite problems with academic studies, the nursing profession is seen as attractive and there is no intention to drop out	Describes a constant search for knowledge and evidence, connecting this to continuing development as a professional nurse and as a human being	Starting to work as a nurse soon is both scary and great fun. Describes a desire to continue studying but first wants to work to feel a bit more secure in the profession

Finally, an overarching main theme of professional development was abstracted from the main categories and shared patterns, Table 2. To ensure trustworthiness data was discussed among the authors in every step of the analysis until consensus was reached [22]

**Table 2:** Example of matrix including two participants, showing how individual and shared patterns developed

	First interview		Second interview		Third interview		Fourth interview	
Student	No. 8	No. 26	No. 8	No. 26	No. 8	No. 26	No. 8	No. 26
Examples subcategories	Help people	Wants to learn	Developed critical thinking	Wants to practice	Seeking evidence	Bad role models	Realizes own limits	Knows how to apply theory
	Possibilities	Manage practical work	Learned a lot	Learn for real	Changed professional view based on knowledge	Negligence	Gained lot of knowledge	Awareness
	Visible result		Changed approach to nursing			Questions the business	Humility	Constantly searching for new knowledge
Example generic categories	Make a difference	Belief in oneself	Theoretically equipped	Need experience	Want to influence	Reality shock	Feeling prepared	Exciting and scary
Main categories	Anticipation		Prepared for internship		Deepened understanding		Insight	
Overarching theme	Ready but not fully trained							

Each column represents an interview with subcategories, generic categories, and main categories. These two participants can represent shared patterns

## Results

One main category was created from interviews performed at the same semester. Results are presented as four main categories: ‘Anticipation’; ‘Prepared for internship’; ‘Deepened understanding; ‘Insight’ with associated generic themes, summarized in the overarching theme: ‘Ready but not fully trained’, Fig. 1.

### Anticipation

In the first interview students expressed their conceptions, expectations and doubts regarding both education and the nursing profession.

#### Make a difference

Students voiced a desire to make a difference, to contribute to society, change structures, and help people. They also voiced expectations of receiving tools to deal with real-life problems and change the world for the better. For some students, this desire to make a difference had meant choosing to end a prosperous career in another field.

*“I have expectations that you should be able to contribute something every day, to feel needed and important, an important cog in the wheel”* (Student no 30).

#### Proud

Students also emphasized they were proud to become nurses and the profession was perceived depicted as incorporating aspects of emotionally rewarding personal development. Employment security was also underscored expressing beliefs that there will always be a need for RNs to help the sick and injured.

*“I am going to have an occupation I’m proud of, when people ask what I do for a living I want to say that I am a nurse and it is the best job in the entire world, that is the strongest anticipation”* (Student no 31).

#### Self-confidence

The students presented a picture of being aware of their own strengths and weaknesses, and expressed a belief in their personal capacity, looking forward to the challenges to come. Students explained the decision to become an RN saying that they had been told they had a suitable personality, but they underscored that the final decision had been their own. Some mentioned having a childhood dream of becoming an RN.

*“Many have told me; you should become a nurse, but it was I who made this decision, because I did not want to become a nurse for a long time because I didn´t want to study”* (Student no 1).

#### Doubts

Doubts were identified in three areas: one´s true nature, life situation, and the nursing profession. Concerns of trying out if RN would be an appropriate occupation, being incapable, coming to dislike the profession or regretting having started the program were linked to ideas about self-awareness and personality. Responsibilities to significant others were mentioned as a potential obstacle in coping with the education. Doubts about the profession concerned poor working conditions including an overwhelming workload, becoming stuck in the middle and not being appreciated.

*“That I shall never be able to relax but will always be sitting in the wrong chair all the time, and to have to struggle financially, no salary, will I be able to take extra shifts or not, yes worries, and if I should feel it isn´t my cup of tea too, it would be sad if I had put a lot of effort into the education”* (Student no 31).

### Prepared for internship

In the second interview after two semesters in theoretical education students were about to put their theoretical knowledge into practical skills.

#### Theoretically equipped

Students expressed a belief in their own progress and having learned a lot, feeling theoretically prepared for internship. However, this experience of being successful in their studies was not particularly influenced by failing exams or not passing a course.

*“A lot of theoretical knowledge that I think is still present even if you forget a little here and there, I think it is in the back of your head somewhere, and anyway, still you don’t feel that you’re thrown in at the deep end, now you get to learn to swim, but you still have some knowledge, that you hopefully will be able to try”* (Student no 11).

#### Need experience

The transition of knowledge from abstract to concrete was expressed as a longing to develop clinical skills. Consequently, the desire for internship was regarded as a long-awaited opportunity to develop practical experience.

*“I’m just looking forward more and more to internship and to see how it is for real and so on, because sometimes you want to be able to see if it is as good as you read in the books”* (Student no 20).

#### Professional approach

Students testified to the experience of having changed their outlook on their personal life and of being able to act professionally when needed. They felt more competent having acquired more knowledge. Students talked about an increased interest in searching for evidence-based practice in daily life and compared this with how they used to do things because they had heard or read about it somewhere. They described this in terms of a change in mindset, being more thorough when it came to searching for information.

*“I have learned the difference between just being nice and being too much sugar and spice with the risk of being condescending, just think of small things such as saying that, like not to say, come on, let’s go and shower, because it may not actually be the way to talk to someone, it becomes a little statement of stupidity, sweetie or love or whatever, so I think my communication has probably improved, and I think I have a slightly more humble attitude than before”* (Student no 3).

#### Trust in educators

A trust in educators emerged in this interview, expressed by expectations about being taught what they need to become an RN. Additionally, sometimes the understanding and relevance of topics brought to the fore in the teaching did not occur until after having completed a course; during the course the aims and structure sometimes came across as unclear and ambiguous. Students expressed taking for granted that all the topics taught built on evidence-based practice.

*“It’s hard to say because I have not practiced it, I feel that we get the tools at university that equip us to think safely”* (Student no 8).

#### Insecurity

Insecurity was brought up by students in terms of lacking knowledge, competence, or ability. The fear of making mistakes and harming a patient was one of the students’ worst fears. Feelings of underperforming were mentioned and often related to personal issues. When talking about pressures felt in relation to studying, students mentioned that they had themselves to blame, for example because of poor planning or prioritizing other things besides studying.

*“The thought of internship makes me incredibly nervous, but also very excited so there are conflicting emotions… it is nerve-racking in both directions, but I hope that you feel safe then with all the theory that we have received, we have gained a very solid knowledge so that’s not what I’m unsure of, it’s rather my own competence to take in everything I’m a little unsure of”* (Student no 23).

#### Fairy-tale world

Since students were about to embark on their clinical practice, issues about this reality were highlighted. There was a preconception of what to expect, combined with thoughts of being taught about a fairy tale world. Even so, the pre-understanding of how things worked was considered valuable. Additionally, an awareness of being underpaid as an RN was raised, but this was overruled by the prospect of receiving emotional rewards in the profession.

*“These things are repeated all the time, but you never get the chance to discuss what reality looks like, and what do I do if it doesn´t look like this, because it doesn´t, so it becomes very much like what the fairy tale world looks like, and we hope reality looks like that too “* (Student no 5).

### Deepened understanding

The third interview took place halfway through the program when all students had attended internship. The internship was described as an opportunity to transfer theoretical knowledge to practical skills. Students particularly testified to experiences involving broadened knowledge of the nursing profession, nurse core competencies and the health-care organization.

#### Constant process

Development at this stage was experienced as challenging. Learning was described as a personal responsibility, although students also stressed the importance of being able to observe supervisors as good role models.

*“It is a constant learning process, searching for knowledge, you should always question why do we do this, there should be evidence… we develop all the time”* (Student no 26).

#### Contrariwise learning

Students described examples of supervision settings in which the tutor did not follow guidelines or failed to uphold patient safety. Students dealt with such ‘negative learning experiences’ by setting personal objectives not to act as these tutors. Students underscored that those experiences of negative examples could potentially be more beneficial given opportunities to reflect on these together with a peer.

*“It is very educational to be out and see both good and bad examples because then you see how you absolutely don´t want to work and how you don´t want to become and what you don´t think is okay”* (Student no 16).

#### Want to influence

When talking about negative matters in health care, students highlighted their visions of making influence. Students talked about the ability to act professionally during internship, something that became complicated when supervisors took shortcuts or skipped guidelines. Sometimes this led to a complex change in mindset about the professional role as students did not want to question the supervisors openly.

*“Sometimes it has also been like this:’You see that I have gloves on me, ok’, although they don´t have gloves,’for educational purposes I have gloves, you know now, okay’.“* (Student no 25).

#### Reality shock

Some clinical placements were described as being charged with a negative atmosphere including staff who acted negligently or carelessly. It was concluded that the nurse core competencies were an excellent theoretical model, but some of them were regarded impossible to maintain. For example, students’ estimations of patients’ safety in clinics ranged from dangerous to secure. An unsafe environment was explained as a consequence of exhausted staff and lack of time. Students were annoyed with the amount of documentation and non-appropriate software, stealing time from patient care. Instances of hierarchical thinking were mentioned as a minor problem, primarily experienced in meetings with employees close to retirement age.

*“You want so incredibly much, and you have your high ambition when you go out [in the clinic] and so, and then there is no time or something, it was kind of quite scary to come out and see how it actually is in reality”* (Student no 10).

#### Pressure

Learning practical skills was described as exhausting since trained staff offered diverse methods, sometimes lacking in evidence. Students also voiced problems identifying ‘the correct way’ to perform medical technical. Additionally, students did not want to oppose their supervisors but rather sought their guidance and support.

*“It was tough to have such a long internship, it was absolutely dreadful … and then you are new and should be so damn alert… because it is our own responsibility, our own learning of course, you want so much… I was actually completely exhausted”* (Student no 33).

### Insight

In the fourth interview, the students were aware that their education was coming to an end, and they were about to leave the student role. Students highlighted that even if they felt prepared to work as RN:s, they did not consider themselves fully trained.

#### Feeling prepared

Students expressed having learnt the professional role, being equipped, and having trust in their own competence and ability. Competence was referred to as a personal capacity, including both professional skills and abilities. Students also testified to having an insight into the necessary knowledge of an RN and expressed gratitude to persistent lecturers pushing them to learn things they initially did not understand.

*“From not really knowing what I got myself into, to knowing I have an ethical compass, and I can question certain actions or prescriptions that I am not sure about, that I wish the patient well, that I can see it”* (Student no 27).

#### Influences of reality

Students reflected on poor terms of employment, mentioning issues of low salary, lack of time and poor working environments. These concerns were strengthened by internship experiences of working alongside tired staff or those only waiting for retirement. Students also reflected on societal values that have a negative impact on RNs, for example increased ethnocentrism, and an overall hardening climate. Moreover, students voiced fears of becoming bored and not caring about their work, linking back to encounters with tired and overworked nurses at the clinic.

*“I saw staff who didn´t have time to eat lunch and such, it may not be a major part of the profession itself but more healthcare in general and that is probably what I have thought about a lot, will it be like that when I finish and have to work for well, thirty more years”* (Student no 13).

#### Exciting and scary

Despite describing a conviction of being able to work as an RN, emotions of insecurity were voiced in terms of an unknown future regarding both graduation and being employed. Worries about ending up in a non-functional workplace were voiced as well as the importance and purpose of collaboration. Having observed teams of varying functionality students also talked about the importance of belonging to a purposeful team. To take on a leading role of an interprofessional team was considered to be both exiting and scary. Additionally, students underscored the advantage of participating in an introductory program when applying for employment, along with a strategy to change workplace if they felt dissatisfied.

*“It feels scary at the same time as it feels really fun and fine to finally finish, I aim for advanced studies eventually, but I want to work to feel a bit more confident”* (Student no 8).

#### Personal development

Students reported an increased level of personal awareness in terms of having developed humility and ability to identify their own shortcomings. Reflections on supervisors working against regulations due to lack of time or laziness, led to reasoning about the risk of ending up the same way, not taking care of patients in an evidence-based and secure way. Another opinion was that the education offered too little time to develop the ability to handle future professional challenges.

*“I have gained a lot of knowledge, but also that you grow a lot as a person and as a human, in what you do you learn a lot about yourself “* (Student no 24).

### Overarching theme: ready but not fully trained

The interviews revealed emotions of being competent but also of lacking knowledge and skills; students considered that continued learning and development was necessary to work as a professional RN. The students pointed out that they would give up working as an RN if they felt there was no need for further learning, because there will always be room for improvement, leading to the overarching theme; Ready but not fully trained as students explained that the nursing profession implies lifelong learning and lifelong improvement.

## Discussion

Findings from this study describe students perceived gradual transformation of becoming a professional RN. In particular our findings indicate that students entering higher education sometimes have a naive or idealized view of the nursing profession, driven by dreams such as helping people, developing a career and delivering excellent nursing, as found by ten Hoeve et al. [30]. Similar to Lindberg et al. [31] we found that today’s entrants to nursing education believe in themselves and their ability to make an impact.

During the first year students were primarily focused on acquiring theoretical knowledge to develop a solid basis. After having completed a year of theoretical education, they felt prepared to test their theoretical knowledge in practice. Moreover, due to the first theoretical year, students’ self-esteem had increased even if they were not always aware of their progress until they had had time to reflect and look back. Their own professional development became tangible when they started to compare their current skills and competence with the experiences they had at the outset of education, a “delayed” professional understanding. Our findings advance earlier work on delayed understanding that refers to a situation in which students taking a particular course have difficulty understanding topics introduced in the teaching, and why particular things should be learned [32]. Later on, they do see the point of taking the course, but this understanding can sometimes be substantially delayed, which may cause some students to experience considerable frustration.

When entering clinical practice students are theoretically equipped, but most students lack practical experience, they are on a Novice level [11]. In the second year, after practicing their skills in clinical environments, students described how theory and practice were joined to become a whole. Our study indicate that the students’ expectations constituted motivation for theoretical studies, which in turn provided readiness for practical training with the support of acquired theoretical knowledge. The experiential learning in clinic seems to give the students possibility to enter the level of Advanced beginner [11].

The clinical environment was sometimes a rough wake-up call when students’ experiences did not quite meet their dreams and expectations, previously described by ten Hoeve et al. [30]. Even so, this study revealed that students testified to experiences of having been able to train according to guidelines, although the staff worked according to routines without practicing person-centered care. Students’ professional development became obvious when they reflected on, and assessed, the behaviors and actions of the clinical staff. Interestingly, students used the experience of meeting supervisors lacking in professional competency as “bad examples” which led to a personal goal not to act in a similar way. From these observations it is clear that learning occurs in all areas, also when there are discrepancies and gaps [33]. Despite the negative experiences of students, our findings suggest that students did mature as human beings, identifying changes in their own behavioral patterns, indicating they were involved in a transformative learning process [34]. Gaining experience from both work and personal life seemed to be beneficial to professional development. Furthermore, during the clinical education, students deepened their understanding through experiential learning and at the time of graduation, the students had developed a professional insight. Benner describes that newly graduated nurses are mostly Advanced beginners [11], but our results suggests that many students have reached the level Competent.

Students professional development became visible in how they transformed their understanding of the six core competencies: person-centered care, evidence-based practice, teamwork and collaboration, safety, quality improvement and informatics [10]. In the early interviews, students spontaneously ranked the importance of each competency, some considered more essential and others less significant. At the end of the program students described a pattern of connection between all core competencies, implying that if one was missing it would be to the detriment of the others. Students’ ability to connect the core competencies as parts forming a coherent whole revealed their understanding of the complexity of the nursing profession as described by Guaroni et al. and Lindberg et al. [31, 35]. Similar to findings reported by Manninen et al. [36] our results showed students’ focus on professional development shifting from managing medical-technical skills to the capability of using a person-centered and holistic perspective when interacting with patients. Our findings of students’ ability to overview the context and achieve a holistic understanding of the core competencies reveals a clear progression in their professional development. The findings presented here indicate that students experience insecurity at the end of their education as they realize they have to master nursing care as well as be able to co-ordinate the professional team, an observation which has previously been reported by Lindberg et al. [31]. These dichotomous emotions of excitement and fear at time of graduation could be connected to the fact that the biggest step from being a student to a professional RN involves taking independent responsibility as shown by Kumaran and Carneys [37]. Additionally, students’ observation that an RN always needs further development clearly indicate that the interviewees reflected on their own professional competence as well as nursing competence in general. This is an important reminder for employers and managers to sanction time for staff professional development activities.

### Strengths and limitations

This study offered longitudinal insights into a sample of 34 students followed throughout their education. Different measures were taken to enhance trustworthiness including dependability and credibility [23]. All students who had entered the nursing program, regardless of previous experiences, were invited to participate. The same interview guide was used on each occasion although students started their studies at different times. All interviews were carried out by the first author, who had a pre-understanding working as a lecturer at the university. To reduce impact on students’ participation no interviews were performed in the semester when the first author graded the students. The interviews took place at one specific university and these contextual boundaries need to be taken into consideration in assessing the transferability of the results to other contexts. However, the Swedish nursing program is regulated by national guidelines, likewise to nursing education globally which suggests that these findings may be of relevance to programs of a similar kind. The findings presented here make important connections the more general concept of authenticity and Benner´s [11] nursing theory and Mezirows theory of transformative learning [34] which may potentially enhance the generalizability of the results.

## Conclusion

The process of developing a professional competence as a nurse progresses gradually. Upon graduation, students feel ready but not fully trained, which signifies a professional approach in which an RN can always improve academic and clinical skills. A solid theoretical knowledge contributes to students reflecting on both their own role and the nurse’s role in clinical settings. Our findings indicate a discrepancy between the content of the theoretical education and the clinical settings since students identified a lack of evidence-based practice. It is clear that students could benefit from increased collaborative work between clinical supervisors and faculty staff at the university.

## Supplementary Information

**Additional file 1.** Interview guide.

## Data Availability

The datasets generated and/or analyzed during the current study are not publicly available due to that the material may contain details about participants that might need to be anonymized but are available from the corresponding author on reasonable request.
