# Supplementary material for: Undergraduate nursing students’ experiences of becoming a professional nurse: a longitudinal study
Source: BMC Nurs. 2022 Aug 6;21:219. doi: 10.1186/s12912-022-01002-0 (PMC9357313; doi:10.1186/s12912-022-01002-0)
Supplement: Supplementary file 1 — Additional file 1. Interview guide. [file 12912_2022_1002_MOESM1_ESM.docx]

Interview guide

**Interview 1**

1) Describe why you want to become a nurse.

2) Did anything affect you to make the choice? A person, an incident or something similar?

3) What are your expectancies on the education?

4) What are your expectancies on the nursing profession?

5) Do you have any misgivings about the education?

6) Do you have any misgivings about the nursing profession?

7) Do you have any doubts about the education?

8) Do you have any doubts about the nursing profession?

9) We have talked about why you want to become a nurse, expectations, ideas and fears before education and about future profession, is there anything you would like to add about these things?

**Interview 2**

1) How do the studies work for you? The structure of education, stress, challenges?

2) What do you think of when you hear the expression “the nurse’s core competence”?

3) How do you perceive your own development regarding the nurse’s core competencies?

Person-centered care, evidence-based practice, teamwork and collaboration, safety, quality improvement and informatics

4) Describe your thoughts about the nursing profession, do you feel that your perception has changed during the first semesters?

5) Tell me how you experience your development towards your future professional role as a nurse?

6) What factors do you perceive affect your development? Education? Study environment? Learning style? Personality? Community development? Privat matters?

7) During the first interview, I asked if anything or anyone influenced you to apply for a nurse. This was answered with thoughts about incidents and people in the personal sphere. Do you think, in retrospect, that events in the outside world may have influenced you to apply for the nursing program? If so, how?

**Interview 3**

1) What are your experiences of feedback, during theoretical courses and during clinical training? Differences and similarities?

2) What significance do you think feedback has for your learning? In theoretical courses and clinical training; differences, and similarities?

3) What is your experience of using reflection in your education? In theoretical courses and clinical training; differences, and similarities?

4) What significance do you think the reflection has for your learning?

5) What are your experiences regarding reflection and your participation in this study? Has it affected you in any way? If yes, how?

6) What is your view of the nurse's core competencies today?

Person-centered care, evidence-based practice, teamwork and collaboration, safety, quality improvement and informatics.

Have your thoughts been influenced by your time in clinical practice, if so, how?

7) We have talked about your experiences of the nurse's core competencies in the clinic, about your views on feedback and reflection, is there anything you would like to add about these things?

**Interview 4**

1) What are your thoughts on the nursing profession?

2) How do you see yourself as a soon-to-be registered nurse?

3) How do you perceive your own professional development during the education?

4) How do you perceive the nurse's role in the interprofessional teamwork?

5) What role do you experience that other in the interprofessional team perceive that the nurse has?

6) What are your experiences of interprofessional teamwork and the role of nurse? Positive, negative experiences?

7) Has the interprofessional teamwork affected you during your education and if so, how?

8) How do you think about the core competencies now that you will soon be a nurse?

Person-centered care, evidence-based practice, teamwork and collaboration, safety, quality improvement and informatics

9) Now that we've talked about your role as a nurse and your own experiences and experiences of interprofessional collaboration, is there anything you would like to add about these issues?
